# The advanced lung cancer inflammation index has an L-shaped association with prognosis in American adults with metabolic dysfunction-associated fatty liver disease: a cohort study

**DOI:** 10.3389/fnut.2025.1571511

**Published:** 2025-07-07

**Authors:** Yuexia Lu, Shuaipeng Yuan, Huazhao Xu, Jiqi Ouyang, Jinsheng Dong, Xin Jiang, Xiao Shao, Runshun Zhang

**Affiliations:** ^1^Guang 'anmen Hospital, China Academy of Chinese Medical Sciences, Beijing, China; ^2^Institute of Basic Research in Clinical Medicine, China Academy of Chinese Medical Sciences, Beijing, China

**Keywords:** MAFLD, ALI, FLI, NHANES, mortality

## Abstract

**Background:**

Regular monitoring and follow-up of patients with metabolic dysfunction-associated fatty liver disease (MAFLD) are of paramount importance in ensuring effective management of the condition. The ALI was assessed as a composite measure reflecting nutritional status and systemic inflammation. It was calculated as body mass index (BMI) (kg/m^2^) × serum albumin (g/dL)/neutrophil-to-lymphocyte ratio (NLR). Our study aims to find the relationship between advanced lung cancer inflammation index (ALI) levels and the prognosis of patients with MAFLD and to determine the predictive value of ALI in this context.

**Methods:**

Multivariate-adjusted Cox regression models were used to analyze the association between ALI and all-cause, cardiovascular, cancer, and diabetes-related mortalities in patients with MAFLD. Kaplan–Meier curves showed the association of ALI with all-cause and cardiovascular mortalities in patients with MAFLD. Follow-up time for this study was calculated from the date of examination to the date of death or to 31 December 2019, and mortality was ascertained using the International Classification of Diseases, 10th Revision codes. Restricted cubic spline (RCS) analysis was conducted to assess the potential non-linear relationship between ALI level and MAFLD prognosis. The predictive ability of ALI was observed using receiver operating characteristic (ROC) curves. Stratified and sensitivity analyses were used to enhance the reliability and robustness.

**Results:**

This study included 2,908 patients with MAFLD from the National Health and Nutrition Examination Survey (NHANES) database between 2003 and 2018. The median follow-up period for the 2,908 participants was 10.3 years, during which 636 deaths occurred. In the Cox regression model, the HRs (95%CIs) for all-cause, cardiovascular, cancer, and diabetes-related mortalities in the last quartile compared to the first quartile of ALI levels were 0.62 (0.44–0.85), 0.25 (0.14–0.45), 0.96 (0.51–1.81), and 0.69 (0.25–1.92), respectively. RCS analysis demonstrated a L-shaped non-linear association between ALI levels and both all-cause and cardiovascular mortalities in participants with MAFLD. Subgroup analyses highlighted population heterogeneity in the relationship between ALI and MAFLD prognosis. ROC curve analysis showed that ALI had strong predictive power for all-cause and cardiovascular mortalities, with area under the curve values of 0.80 (0.77–0.83) and 0.82 (0.74–0.89), respectively.

**Conclusion:**

There was an L-shaped nonlinear association of the protective effect of ALI: when the indicators are below specific thresholds (all-cause mortality 71.48, cardiovascular mortality 68.54), a higher ALI was significantly associated with reduced mortality risks in MAFLD patients; otherwise the protective effect tended to be consistent. ALI exhibits a robust predictive capability for all-cause and cardiovascular mortalities among participants with MAFLD, providing a valuable prognostic tool for optimizing patient management. We recommend early surveillance and management of patients with MAFLD to improve patient survival.

## 1 Introduction

Originating in 2020, metabolic dysfunction-associated fatty liver disease (MAFLD) was conceptualized to supersede the traditional term: non-alcoholic fatty liver disease (NAFLD). In 2023, metabolic dysfunction-associated steatotic liver disease (MASLD) was introduced as an updated nomenclature. A study showed (1) significant concordance across NAFLD, MAFLD, and MASLD classifications.

MAFLD is a widespread liver disease of significant global concern, affecting approximately one-third of the global population, with an increasing prevalence (2). In addition to liver-specific morbidity and mortality, MAFLD is associated with an elevated risk of extrahepatic conditions, including type 2 diabetes mellitus (T2DM), extrahepatic cancers (notably colorectal cancer), cardiovascular disease and chronic kidney disease. Therefore, this disease places a heavy burden on global health (3, 4). Identifying reliable prognostic markers for MAFLD is critical for enabling early intervention and effective disease management.

Chronic inflammatory responses have been identified as pivotal pathophysiological mechanisms that drive MAFLD initiation and progression, potentially through oxidative stress and insulin resistance (5–9). Persistent inflammation can exacerbate progressive extracellular matrix (ECM) deposition, a process closely associated with adverse outcomes such as liver failure, hepatocellular carcinoma, and death (10). Serum albumin is a key marker of nutritional status. Serum albumin level represents a potential biomarker for early hepatic failure and a possible predictor (11). Therefore, the prognosis of MAFLD patients is related to multiple factors. The advanced lung cancer inflammation index (ALI) combines the status of inflammation, albumin levels, and BMI levels, and can comprehensively assess their impact on the outcomes of MAFLD patients. Previous studies have also confirmed the prognostic value of ALI in chronic diseases.

Initially developed for evaluating the prognosis of lung cancer (12, 13), ALI has since been applied to other conditions, including hypertension (14), coronary artery disease (15), and various malignancies (16). Despite ALI’s widespread use, no study to date has explored its relationship with the prognosis of those with MAFLD. And our research has worked on this.

## 2 Materials and methods

### 2.1 Study population

**Figure 1:**
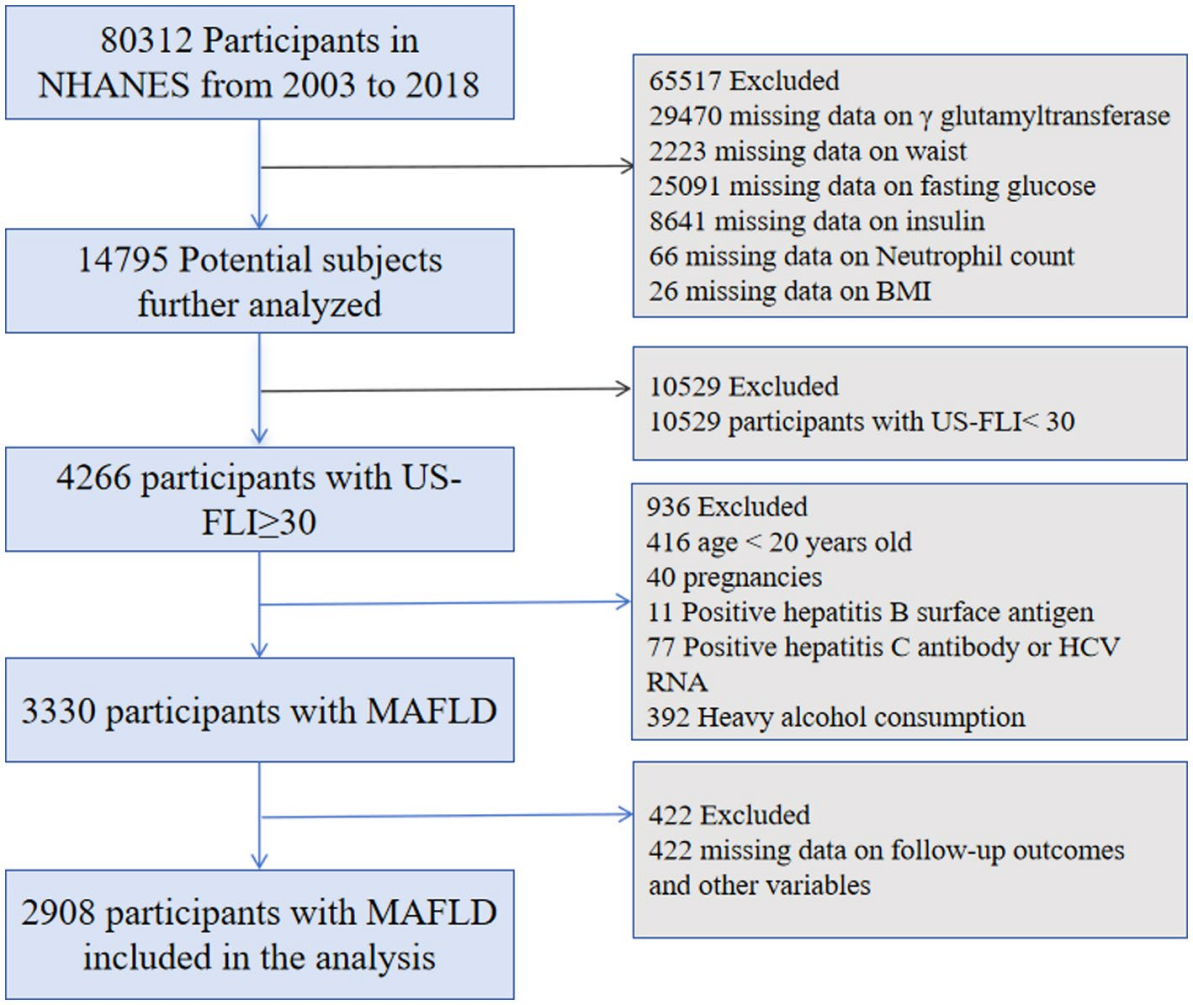
Flowchart depicting the process of selecting the study population.

This cohort study used data from the National Health and Nutrition Examination Survey (NHANES), which employs a stratified, multistage probability sample strategy to perform cross-sectional assessments of the health and nutritional status of the American people. An ethics review board from the National Center for Health Statistics authorized the NHANES protocol. Our analysis collected data from eight survey cycles, spanning 16 years, from 2003 to 2018. The procedure for including and excluding data is shown in Figure 1.

### 2.2 ALI scoring

**Figure 2:**
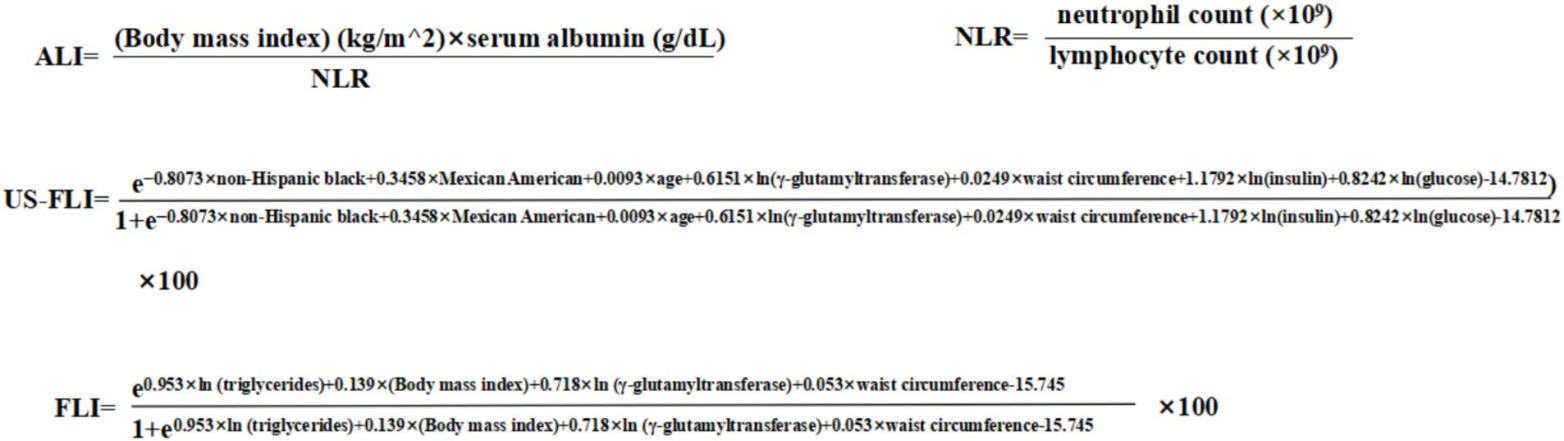
The formula for ALI, NLR, US-FLI and FLI.

ALI is a composite score that reflects both inflammation levels and nutritional status. A high value of ALI suggests a low level of inflammation and good nutritional. The formula for ALI is provided in Figure 2. The formula includes neutrophil-to-lymphocyte ratio (NLR), which is an indicator of inflammation.

### 2.3 MAFLD diagnosis

Liver biopsy is the best way to diagnose MAFLD. However, its invasiveness, high cost, and potential complications limit its wide application (17, 18). Thus, non-invasive markers provide a more practical diagnostic approach. In this study, we employed the United States fatty liver index (US-FLI), a non-invasive marker developed and validated using the NHANES database, with a diagnostic area under the curve (AUC) of 0.80 (19). A US-FLI score of ≥30 was considered to have hepatic steatosis. The third equation in the Figure 2 shows the equation of the US-FLI. To verify the robustness of our results, we performed a sensitivity analysis using a widely validated marker: the Fatty Liver Index (FLI), with a diagnostic accuracy of 0.84 (20). An FLI score ≥60 was considered indicative of hepatic steatosis. The fourth equation in Figure 2 demonstrates the equation of the FLI.

### 2.4 Follow-up and outcomes

The mortality outcomes and classification were determined by linking to the National Death Index (NDI) records. The NDI provides detailed mortality data using standardized codes based on the International Classification of Diseases, Tenth Revision (ICD-10). The follow-up period was calculated from the date of examination to the date of death or the end of the follow-up period. The follow-up of participants in this study continued until December 31, 2019.

### 2.5 Covariates

**Figure 3:**
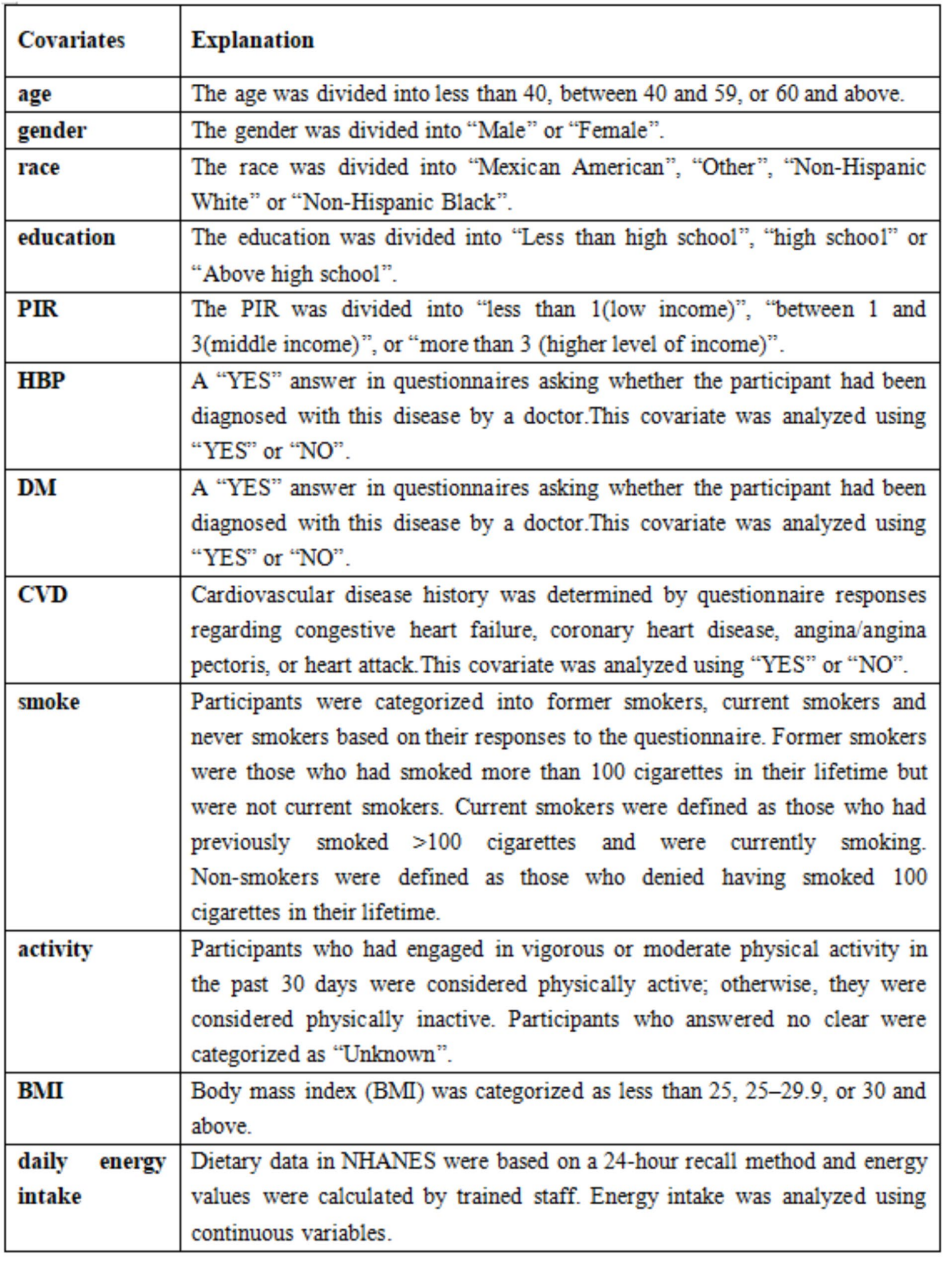
The details of the covariates.

Covariates were selected based on previous studies (21). These covariates included demographic characteristics [race, sex, age, education, and poverty-income ratio (PIR)], laboratory tests [high-density lipoprotein (HDL)-C levels, alanine transaminase (ALT), aspartate aminotransferase (AST), triglycerides (TG), and total cholesterol (TC)], other diseases [diabetes (DM), cardiovascular disease (CVD), and high blood pressure (HBP)], and behavioral factors (physical activity and smoking). Daily energy intake and Body mass index (BMI) were also included. Data from laboratory tests were analyzed as continuous variables, and the descriptions of the remaining covariates are shown in Figure 3. Age and PIR were grouped according to previous studies (22, 23). The cut-off values of BMI were determined based on the criteria of the World Health Organization (WHO).

### 2.6 Statistical analysis

Categorical variables were represented as percentages (%), while continuous variables were summarized using the mean (standard error) or median (interquartile range). For data analysis, we used the chi-square test for categorical variables, t-test for normally distributed data, and Wilcoxon rank-sum test for skewed data. Weighted Cox proportional hazards models were employed to evaluate the association between ALI and mortality outcomes, including all-cause, cardiovascular, cancer, and diabetes-related mortalities. Kaplan–Meier survival curves were generated to visually depict the link between ALI and all-cause and cardiovascular mortalities.

To investigate if ALI and cardiovascular and all-cause deaths have a non-linear connection among people with MAFLD, we performed restricted cubic spline (RCS) testing. Subgroup analyses, stratified by education, race, sex, age, diabetes, hypertension, and cardiovascular disease, were conducted to examine the association between ALI and all-cause and cardiovascular mortalities in specific populations. For visual clarity, ALI values were rescaled to ALI/10 in the subgroup analyses. Finally, sensitivity analyses were performed to view the stability of the findings under various data processing scenarios.

The data was processed and analyzed using R version 4.3.0 and the Storm Statistical Platform. The threshold for statistical significance was *p* < 0.05.

## 3 Results

### 3.1 Baseline characteristics

The initial dataset included 80,312 participants. Those with missing data on ALI and US-FLI (*n* = 65,517), US-FLI scores <30 (*n* = 10,529), minors (*n* = 416), pregnant women (*n* = 40), individuals with hepatitis B (*n* = 11) or hepatitis C (*n* = 77), excessive alcohol consumption (*n* = 392), and missing survival outcomes or covariates (*n* = 422) were excluded, leaving 2,908 patients with MAFLD (Figure 1).

Participants were stratified into four quartiles based on ALI values: Q1 (ALI ≤ 49.53), Q2 (49.53 < ALI ≤ 68.54), Q3 (68.54 < ALI ≤ 90.99), and Q4 (ALI > 90.99). In Table 1, we can see that compared with participants in the lower ALI quartiles, those in the higher ALI quartiles were older, less likely to be obese, and had fewer non-Hispanic White individuals. They also had higher education levels, greater energy intake, and a lower prevalence of hypertension, diabetes, and cardiovascular diseases. Participants who had never smoked were more likely to have higher ALI values. The levels of HDL-C, ALT, AST, TG, and TC, as well as physical activity, were similar across the ALI quartiles, and the poverty-income ratio showed no significant differences among the groups.

**Table 1:** Baseline characteristics of the participants (*N* = 2,908).

Characteristics	ALI				*p*
	Quantile 1	Quantile 2	Quantile 3	Quantile 4	
	<49.53	49.53–68.54	68.54–90.99	>90.99	
Participants, n	727	727	727	727	
Energy intake, median (IQR), kcal/day	1780 (1364.50–2409.50)	1908 (1419.50–2534.50)	1951 (1397–2,533)	1992 (1460–2,624)	0.006
HDL-C, median (IQR), mg/dL	46 (39–54)	44 (38–52)	44 (38–51)	44 (38–52)	<0.001
ALT, median (IQR) U/L	23 (18–31)	25 (20–34)	27 (21–37)	27 (21–39)	<0.001
AST, median (IQR) U/L	24 (20–28)	24 (20–29)	25 (21–30)	26 (22–32)	0.004
TG, median (IQR), mg/dL	132 (94–193)	144 (105–202.5)	151 (108–202)	147 (101–218.5)	0.004
TC, median (IQR), mg/dL	186 (161–220.5)	196 (170–222)	195 (170–221)	199 (173–226)	<0.001
Gender, *n* (%)					<0.001
Male	58.84	63.37	58.40	49.81	
Female	41.16	36.63	41.60	50.19	
Race, *n* (%)					<0.001
Mexican American	8.65	11.20	13.90	14.91	
Other	9.91	8.51	10.00	8.94	
Non-Hispanic White	78.89	76.48	70.28	64.05	
Non-Hispanic Black	2.55	3.81	5.81	12.10	
Education, *n* (%)					0.023
Less than high school	23.35	21.19	21.21	23.19	
High school	29.59	22.93	26.43	23.58	
Above high school	47.06	55.88	52.36	53.23	
Hypertension, *n* (%)					0.011
Yes	55.38	50.36	47.60	47.73	
No	44.62	49.64	52.40	52.27	
CVD, *n* (%)					<0.001
Yes	19.62	10.92	10.42	9.35	
No	80.38	89.08	89.58	90.65	
DM, *n* (%)					0.046
Yes	19.70	19.54	17.64	14.21	
No	76.48	76.98	79.75	81.36	
Unknown	3.82	3.48	2.61	4.43	
Smoke status, *n* (%)					<0.001
Former	37.23	32.18	28.19	29.00	
Never	41.36	49.90	53.63	57.33	
Current	21.41	17.92	18.19	13.67	
Age, *n* (%)					<0.001
≤39	47.42	33.88	27.36	24.43	
40–59	35.93	43.71	42.85	42.88	
≥60	16.65	22.41	29.80	32.69	
PIR, *n* (%)					0.327
<1	44.19	46.38	47.17	42.01	
1–3	40.85	41.63	39.37	43.56	
>3	14.97	12.00	13.47	14.43	
BMI, *n* (%)					<0.001
<25	54.14	66.79	80.08	80.87	
25–29.9	35.75	30.00	17.74	17.86	
≥30	10.12	3.21	2.18	1.27	
Physical activity, *n* (%)					0.008
Inactive	60.54	51.22	56.34	59.06	
Active	38.79	48.30	43.31	40.77	
Unknown	0.67	0.48	0.35	0.18	

Continuous variables not conforming to normal distribution were expressed as median (IQR) and compared using the Wilcoxon rank sum test.

### 3.2 ALI and mortality in patients with MAFLD

The median follow-up period for the 2,908 participants was 10.3 years, during which 636 deaths occurred, leading to a mortality rate of 21.87%. Among these, there were 166 cardiovascular, 154 cancer, and 49 diabetes-related deaths.

As shown in the Table 2, after adjusting for all covariates, compared with the Q1 group, the hazard ratios (HRs) (95% CI) for all-cause mortality in the Q2, Q3, and Q4 groups were 0.70 (0.54–0.91), 0.59 (0.45–0.78), and 0.62 (0.44–0.85), respectively. This indicated that with each unit increase in ALI, the risk of all-cause mortality decreased by 30, 41, and 38% in the Q2 to Q4 groups. For cardiovascular mortality, the HRs (95% CI) were 0.48 (0.28–0.81), 0.46 (0.27–0.77), and 0.25 (0.14–0.45), indicating reductions of 52, 54, and 75%, respectively, compared with Q1. ALI exhibited a stronger positive effect on cardiovascular mortality than on all-cause mortality. ALI level association with cancer or diabetes-related mortalities was not statistically significant. Consequently, subsequent analyses focused on all-cause mortality and cardiovascular mortality.

**Table 2:** The relationship between ALI and all-cause, CVD, cancer, and DM-related mortality.

ALI	Model 1	Model 2	Model 3
	HR (95%CI)	HR (95%CI)	HR (95%CI)
All-cause mortality			
Quantile 1	Ref.	Ref.	Ref.
Quantile 2	0.51 (0.39, 0.66)	0.61 (0.47, 0.79)	0.70 (0.54, 0.91)
Quantile 3	0.34 (0.26, 0.46)	0.53 (0.4, 0.7)	0.59 (0.45, 0.78)
Quantile 4	0.32 (0.24, 0.43)	0.53 (0.39, 0.71)	0.62 (0.44, 0.85)
Cardiovascular mortality			
Quantile 1	Ref.	Ref.	Ref.
Quantile 2	0.38 (0.23, 0.63)	0.46 (0.28, 0.76)	0.48 (0.28, 0.81)
Quantile 3	0.33 (0.2, 0.55)	0.49 (0.3, 0.82)	0.46 (0.27, 0.77)
Quantile 4	0.18 (0.1, 0.33)	0.27 (0.15, 0.5)	0.25 (0.14, 0.45)
Cancer-related mortality			
Quantile 1	Ref.	Ref.	Ref.
Quantile 2	0.79 (0.47, 1.3)	0.96 (0.59, 1.59)	1.09 (0.65, 1.83)
Quantile 3	0.51 (0.29, 0.91)	0.83 (0.47, 1.48)	0.94 (0.52, 1.69)
Quantile 4	0.44 (0.24, 0.77)	0.83 (0.46, 1.48)	0.96 (0.51, 1.81)
Diabetes-related mortality			
Quantile 1	Ref.	Ref.	Ref.
Quantile 2	0.33 (0.14, 0.8)	0.36 (0.15, 0.88)	0.49 (0.16, 1.47)
Quantile 3	0.23 (0.08, 0.68)	0.28 (0.09, 0.83)	0.38 (0.11, 1.33)
Quantile 4	0.37 (0.14, 0.96)	0.41 (0.15, 1.14)	0.69 (0.25, 1.92)

HR, hazard ratio; CI, confidence interval; Ref: reference. Model I: adjusted for no covariates. Model II: adjusted for age, gender, race. Model III: adjusted for age, gender, race, education, BMI, PIR, HDL-C, ALT, AST, TG, TC, HBP, DM, CVD, smoking, activity, daily energy intake.

**Figure 4:**
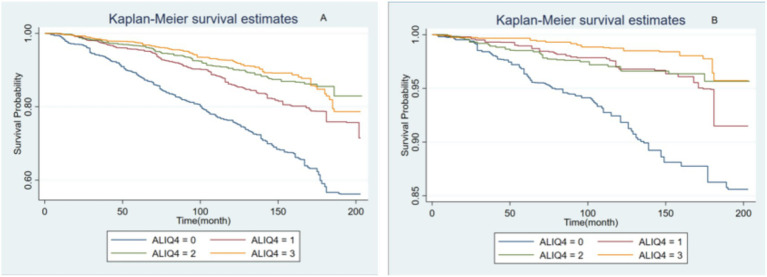
The Kaplan–Meier survival analysis curves for all-cause **(A)** and cardiovascular mortalities **(B)**.

In Figures 4A,B show the Kaplan–Meier survival analysis curves for all-cause and cardiovascular mortalities, respectively. The values 0–3 represent the four ALI quartiles (Q1, Q2, Q3, Q4). All-cause and cardiovascular mortalities were reduced among participants with MAFLD when the ALI was greater than the Q1 level.

### 3.3 Non-linear relationship analysis

**Figure 5:**
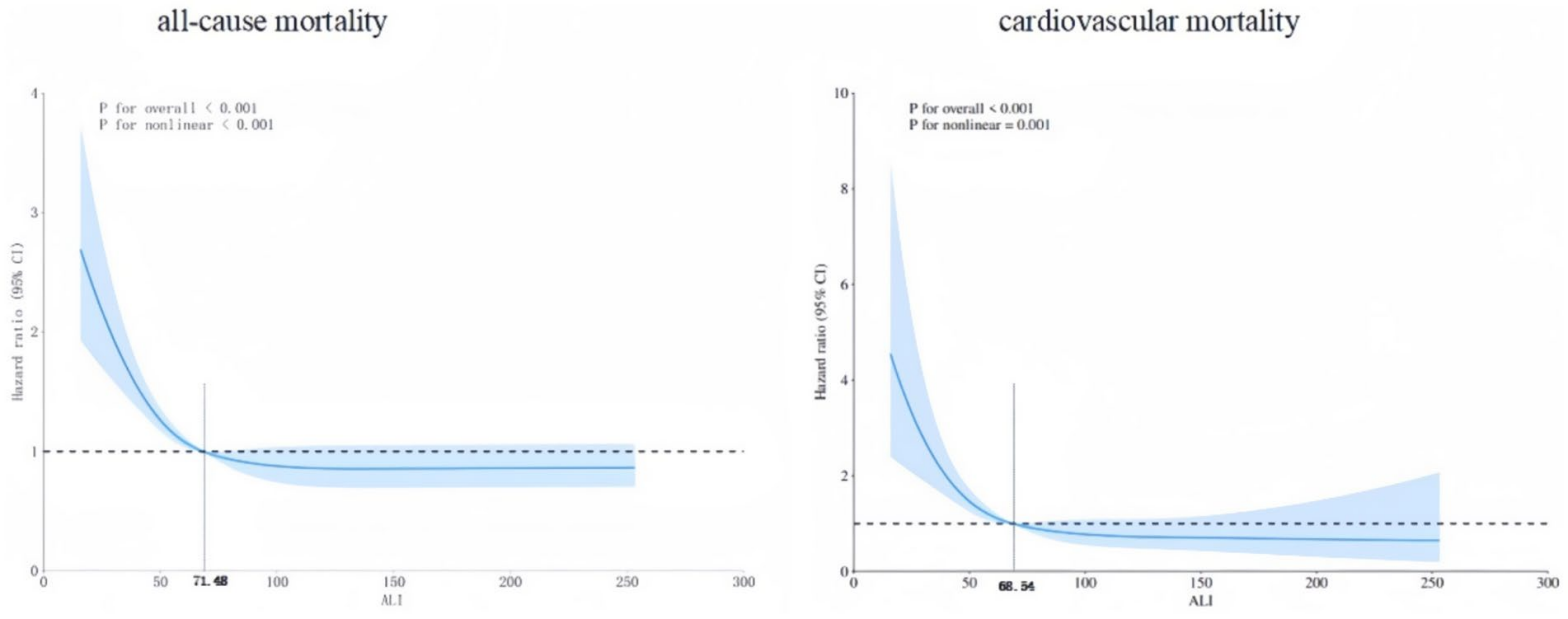
The RCS analysis for all-cause and cardiovascular mortalities.

RCS analysis revealed a distinct L-shaped non-linear relationship between ALI and both all-cause and cardiovascular mortalities (Figure 5). The inflection points for all-cause and cardiovascular mortalities were 71.48 and 68.54, respectively.

### 3.4 Subgroup analysis

**Figure 6:**
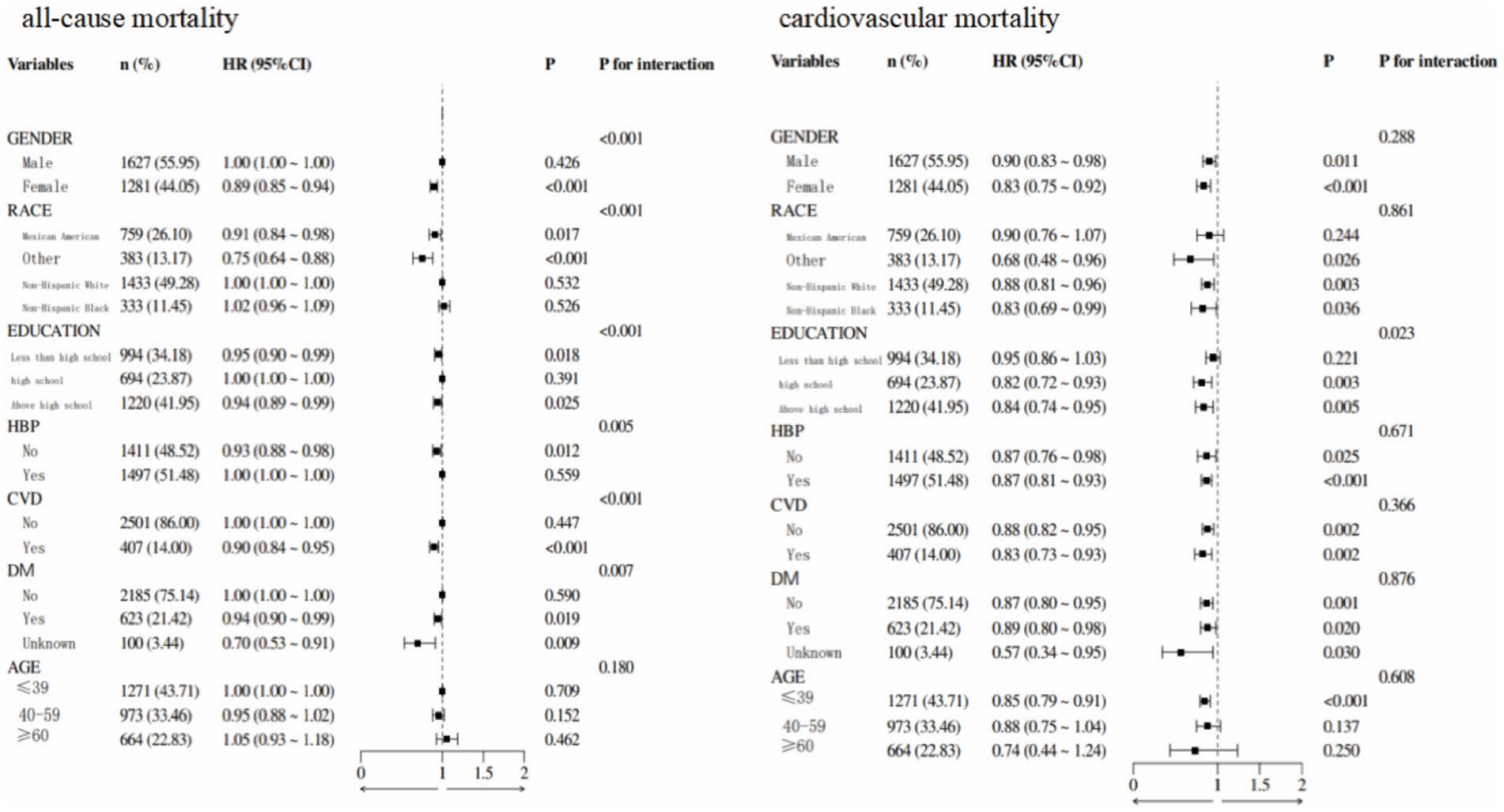
Forest plot demonstrated the relationship between ALI and all-cause and cardiovascular mortalities.

Subgroup analysis showed that in patients with MAFLD, 55.95% were male, 49.28% were aged 20–39 years, 41.95% were non-Hispanic White, and 43.71% had an education level above high school (Figure 6). Stratified analyses by sex, race, education, age, diabetes status, hypertension, and cardiovascular disease revealed significant interactions between ALI and all-cause mortality in all strata, except for age. This finding suggests heterogeneity in the association between ALI levels and all-cause mortality across populations. The correlation between ALI and all-cause mortality in patients with MAFLD was more pronounced in women. In the interaction test for cardiovascular mortality, no interaction *p*-values reached statistical significance, except for education level.

### 3.5 Sensitivity analysis

To confirm the reliability of the results, three additional cox regression analyses were performed (Table 3). Firstly, after excluding extreme ALI values, the association between ALI and both all-cause and cardiovascular mortalities remained significant, with HRs for the Q4 group being 0.62 and 0.25, respectively. Secondly, after excluding participants who died within 2 years of the end of the follow-up period, the results from the Cox regression analyses remained consistent. Lastly, when FLI ≥ 60 was used as the diagnostic criterion, expanding the cohort to 5,397 participants with MAFLD, the results also remained robust. Sensitivity analyses confirmed that higher ALI levels were associated with a reduced risk of all-cause and cardiovascular mortalities in people with MAFLD, whereas no significant effect on cancer or diabetes-related mortalities was observed.

**Table 3:** Result of sensitivity analysis.

ALI	Model 1	Model 2	Model 3
	HR (95%CI)	HR (95%CI)	HR (95%CI)
① After excluding extreme ALI values			
All-cause mortality			
Quantile 1	Ref.	Ref.	Ref.
Quantile 2	0.51 (0.39, 0.66)	0.61 (0.47, 0.79)	0.70 (0.54, 0.91)
Quantile 3	0.34 (0.26, 0.46)	0.53 (0.4, 0.7)	0.60 (0.45, 0.78)
Quantile 4	0.32 (0.24, 0.43)	0.53 (0.39, 0.72)	0.62 (0.44, 0.86)
Cardiovascular mortality			
Quantile 1	Ref.	Ref.	Ref.
Quantile 2	0.38 (0.23, 0.63)	0.46 (0.28, 0.76)	0.48 (0.28, 0.81)
Quantile 3	0.33 (0.2, 0.55)	0.49 (0.3, 0.82)	0.46 (0.27, 0.77)
Quantile 4	0.18 (0.1, 0.33)	0.28 (0.15, 0.5)	0.25 (0.14, 0.46)
Cancer-related mortality			
Quantile 1	Ref.	Ref.	Ref.
Quantile 2	0.79 (0.47, 1.3)	0.96 (0.59, 1.59)	1.09 (0.65, 1.83)
Quantile 3	0.51 (0.29, 0.91)	0.83 (0.47, 1.48)	0.94 (0.52, 1.69)
Quantile 4	0.44 (0.24, 0.76)	0.82 (0.45, 1.49)	0.95 (0.51, 1.81)
Diabetes-related mortality			
Quantile 1	Ref.	Ref.	Ref.
Quantile 2	0.33 (0.14, 0.8)	0.36 (0.15, 0.88)	0.49 (0.16, 1.47)
Quantile 3	0.23 (0.08, 0.68)	0.28 (0.09, 0.83)	0.38 (0.11, 1.33)
Quantile 4	0.37 (0.14, 0.96)	0.42 (0.15, 1.15)	0.69 (0.25, 1.92)
② Excluding participants who died within 2 years			
All-cause mortality			
Quantile 1	Ref.	Ref.	Ref.
Quantile 2	0.52 (0.40, 0.69)	0.63 (0.48, 0.83)	0.73 (0.55, 0.96)
Quantile 3	0.33 (0.25, 0.45)	0.52 (0.38, 0.69)	0.58 (0.43, 0.78)
Quantile 4	0.33 (0.24, 0.45)	0.55 (0.40, 0.76)	0.65 (0.45, 0.92)
Cardiovascular mortality			
Quantile 1	Ref.	Ref.	Ref.
Quantile 2	0.38 (0.22, 0.65)	0.47 (0.28, 0.79)	0.48 (0.28, 0.85)
Quantile 3	0.28 (0.16, 0.48)	0.42 (0.24, 0.73)	0.39 (0.22, 0.69)
Quantile 4	0.16 (0.09, 0.31)	0.25 (0.13, 0.46)	0.23 (0.12, 0.43)
Cancer-related mortality			
Quantile 1	Ref.	Ref.	Ref.
Quantile 2	0.83 (0.47, 1.46)	1.02 (0.59, 1.76)	1.16 (0.66, 2.05)
Quantile 3	0.55 (0.30, 1.01)	0.91 (0.49, 1.70)	1.06 (0.562, 2.00)
Quantile 4	0.54 (0.30, 0.97)	1.03 (0.55, 1.93)	1.26 (0.64, 2.51)
Diabetes-related mortality			
Quantile 1	Ref.	Ref.	Ref.
Quantile 2	0.34 (0.13, 0.88)	0.37 (0.14, 0.97)	0.51 (0.15, 1.75)
Quantile 3	0.26 (0.09, 0.79)	0.31 (0.10, 0.96)	0.42 (0.11, 1.63)
Quantile 4	0.41 (0.15, 1.12)	0.46 (0.16, 1.33)	0.77 (0.26, 2.30)
③ FLI ≥60 was used as the diagnostic criterion			
All-cause mortality			
Quantile 1	Ref.	Ref.	Ref.
Quantile 2	0.45 (0.34, 0.59)	0.53 (0.40, 0.70)	0.55 (0.42, 0.73)
Quantile 3	0.36 (0.27, 0.49)	0.48 (0.36, 0.65)	0.53 (0.40, 0.71)
Quantile 4	0.33 (0.25, 0.44)	0.48 (0.36, 0.64)	0.56 (0.42, 0.74)
Cardiovascular mortality			
Quantile 1	Ref.	Ref.	Ref.
Quantile 2	0.33 (0.20, 0.55)	0.40 (0.24, 0.66)	0.4 (0.24, 0.67)
Quantile 3	0.36 (0.22, 0.60)	0.48 (0.29, 0.81)	0.52 (0.31, 0.87)
Quantile 4	0.23 (0.13, 0.41)	0.32 (0.18, 0.58)	0.36 (0.21, 0.65)
Cancer-related mortality			
Quantile 1	Ref.	Ref.	Ref.
Quantile 2	0.56 (0.33, 0.97)	0.69 (0.40, 1.20)	0.80 (0.45, 1.42)
Quantile 3	0.38 (0.21, 0.68)	0.52 (0.28, 0.96)	0.62 (0.34, 1.13)
Quantile 4	0.52 (0.31, 0.87)	0.82 (0.47, 1.43)	1.10 (0.62, 1.93)
Diabetes-related mortality			
Quantile 1	Ref.	Ref.	Ref.
Quantile 2	0.37 (0.15, 0.95)	0.41 (0.16, 1.05)	0.44 (0.16, 1.21)
Quantile 3	0.45 (0.16, 1.26)	0.54 (0.19, 1.49)	0.62 (0.22, 1.72)

Model I: adjusted for no covariates. Model II: adjusted for age, gender, race. Model III: adjusted for age, gender, race, education, BMI, PIR, HDL-C, ALT, AST, TG, TC, HBP, DM, CVD, smoking, activity, daily energy intake.

### 3.6 ROC curve analysis

**Figure 7:**
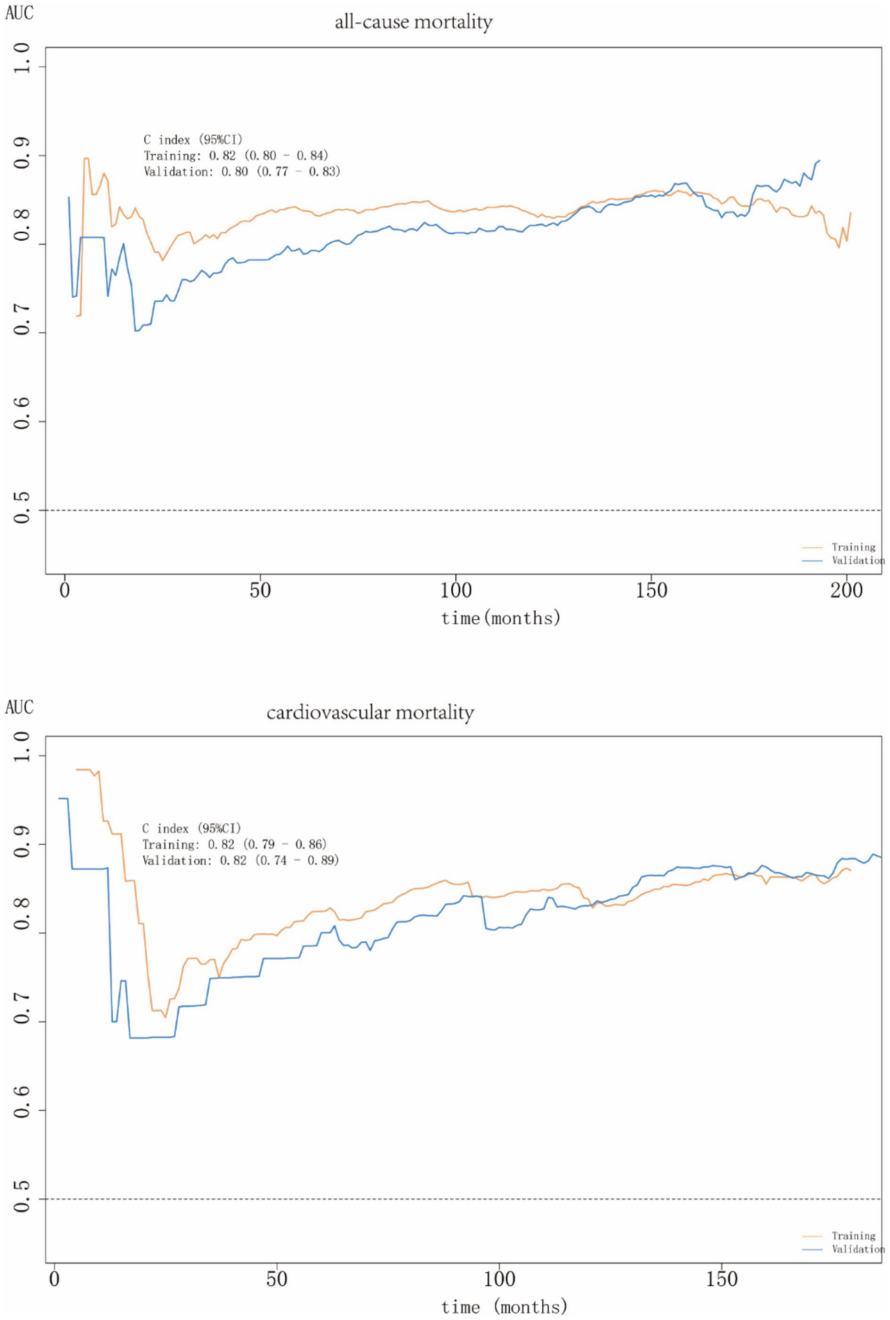
ROC curves of all-cause and cardiovascular mortalities.

ROC curves do not account for time, whereas time-dependent ROC curves evaluate the predictive performance of factors across various time points. These time-dependent ROC curves demonstrated that ALI had excellent predictive accuracy for both all-cause and cardiovascular mortalities in people with MAFLD, with AUC values of 0.80 (0.77–0.83) and 0.82 (0.74–0.89), respectively (Figure 7).

## 4 Discussion

To our knowledge, this is the first large-scale cohort investigation to explore the prognostic relationship between ALI and MAFLD. Our findings demonstrated a significant inverse association between ALI levels and all-cause and cardiovascular mortalities in patients with MAFLD, whereas no statistically significant relationship was observed for cancer or diabetes-related mortalities. RCS analysis revealed a distinct L-shaped non-linear relationship between ALI levels and mortality outcomes. Subgroup analyses revealed population heterogeneity in ALI levels and all-cause mortality among patients with MAFLD. There was no interaction between the subgroups for cardiovascular mortality, except for education level. ROC curve analysis confirmed the strong predictive ability of ALI for all-cause and cardiovascular mortality, with AUC values of 0.80 and 0.82, respectively.

The roles of inflammation and diet in MAFLD pathogenesis have been widely discussed. Gong et al. (24) discovered that systemic inflammation is strongly associated with hepatic steatosis risk. Pro-inflammatory diets are implicated in increased MAFLD risk, prompting public health initiatives to prioritize nutrient-dense food availability (6). Nutritional deficiencies, including protein-energy malnutrition and subclinical micronutrient deficiencies, are linked to hepatic steatosis (25). Moreover, supplementation with vitamins E and D has shown the potential to improve liver function and lipid metabolism and reduce hepatic steatosis risk (26). Among various dietary patterns, the Mediterranean diet stands out for its benefits in reducing cardiovascular risk, hepatic fat accumulation, and fibrosis progression (26). Our study builds upon this foundation, providing further evidence for the interplay between inflammation, nutrition, and prognosis in patients with MAFLD.

Oxidative stress plays a critical role in MAFLD pathogenesis by inducing endoplasmic reticulum stress, increasing pro-inflammatory cytokine release, and activating hepatic stellate cells, leading to fibrosis (26, 27). Approximately 33% of the inflammation-related effects in MAFLD are mediated through the triglyceride-glucose index, highlighting the exacerbating role of insulin resistance in inflammation (28, 29). In contrast, insulin resistance promotes hepatic fat accumulation by increasing free fatty acid flux and hyperinsulinemia-driven anabolic pathways (30). Nutrient-rich foods may play a role in MAFLD prognosis through antioxidant, anti-inflammatory, lipid, and insulin metabolism. Nutrient-rich foods prevent oxidative stress in the liver by reducing glutathione disulfide/glutathione and thiobarbituric acid reactive species in the liver, which can reduce the expression levels of the gene associated with pro-oxidant activity (26). Diet can also balance redox activity by regulating mitochondrial function (31). Nutrient-rich foods may reduce hepatic levels of interleukin (IL)-2, IL-6, and tumor necrosis factorα, alleviating inflammatory injury (32). In addition, nutrient-rich foods may regulate glucose metabolism and modulate and optimize energy metabolism (26).

Several studies have explored the predictive roles of inflammatory and nutritional indicators in the prevalence and prognosis of MAFLD. Zhang et al. (33) identified novel systemic inflammation markers, including the platelet-to-lymphocyte ratio (PLR), NLR, and systemic immune-inflammation index (SII), as predictors of cardiovascular mortalities in MAFLD. This study found that the AUC values of NLR, SII, and PLR were 0.69, 0.60, and 0.52, respectively. Therefore, in comparison, ALI has a higher predictive value, which was also confirmed by another study (34). Serum albumin level represents a potential biomarker for early hepatic failure and a possible predictor (11). Patients with high albumin levels have a lower risk of death or the need for *in situ* liver transplantation (35). ALI, which includes BMI, serum albumin level, and NLR, reflects the interplay between nutrition, immunity, and systemic inflammation. It has demonstrated prognostic value in diverse chronic conditions, including T2DM, hypertension, and kidney disease (36–38). Interestingly, BMI in ALI is theoretically protective against MAFLD, contrary to the belief that obesity adversely affects MAFLD. First, the effect of BMI may explain the L-shaped non-linear correlation observed in the RCS plot. Second, components of ALI, such as inflammation and BMI, interact with each other. Inflammation reduces appetite and leads to malnutrition, which in turn can lead to weight loss (39). Finally, there may be an obesity paradox in the population with MAFLD, where higher BMI may confer survival advantages in terms of nutritional and cardiovascular reserves (40, 41). Some studies have suggested that although overweight patients or patients with obesity are at a higher risk of developing liver disease, they have a survival advantage over lean patients (42, 43). However, we think that high ALI scores resulting from high BMI do not have the same mortality risk reduction benefit as high ALI achieved through moderate BMI. Merely relying on increasing BMI to enhance ALI is not advisable. Weight loss measures should still be reserved for obese patients with high metabolic risks. Sufficient nutritional reserves, low levels of inflammation, and the absence of metabolic burdens brought about by severe obesity better align with the obesity paradox observed in advanced chronic diseases. Future research could stratify ALI by BMI to identify the optimal BMI range.

The L-shape non-linear relationship of RCS analysis has meaningful points: Below the thresholds (71.48 for all-cause mortality and 68.54 for cardiovascular mortality), a higher ALI was significantly associated with reduced mortality risks in MAFLD patients. Above these thresholds, this protective effect plateaued. Specifically, in MAFLD patients, increasing ALI below 71.48 was associated with a significant reduction in all-cause mortality. Similarly, increasing ALI below 68.54 was associated with a significant reduction in cardiovascular mortality. However, when ALI exceeded 71.48 and 68.54, respectively, further increases conferred limited additional benefit for reducing either all-cause or cardiovascular mortality.

Beyond its prognostic value, ALI provides actionable guidance for risk-stratified management of MAFLD: the inflection point for all-cause mortality is ALI = 71.48, and the inflection point for cardiovascular mortality is ALI = 68.54. Considering the actual situation, ALI ≤ 68.54 can be used as a comprehensive high-risk cut-off point. When ALI ≤ 68.54, it is recommended to strengthen follow-up. Secondly, in terms of comorbidity management: the high-risk group (ALI ≤ 68.54) needs to actively carry out secondary prevention of cardiovascular diseases; priority should be given to controlling inflammation. The calculation of ALI is convenient and has a low cost, making it practical for risk monitoring in the MAFLD population. However, it should be noted that: ALI is not an independent decision-making tool and needs to be used in combination with other indicators (such as FIB-4 for assessing liver fibrosis). Secondly, dynamic monitoring of ALI changes is more important than a single value.

Interestingly, the inverse association between ALI levels and all-cause mortality was more pronounced in females. This phenomenon may be related to the inherent biological differences between genders, which profoundly influence the disease process and prognosis. Mechanistically, estrogen exerts a protective effect on liver diseases by reducing oxidative stress, insulin resistance, lipid metabolism, and fibrosis (44–46). The liver is not only a key regulatory organ for metabolic homeostasis but also a major target for estrogen signaling - estrogen receptors *α* (estrogen receptor α, ERα) and *β* (estrogen receptor β, ERβ) bind to estrogen response element (estrogen response element, ERE) to directly regulate lipid and glucose metabolism in the liver (45). Studies have confirmed that a decline in estrogen levels promotes the development of extensive hepatic steatosis and the progression of liver fibrosis (47). Additionally, estrogen has anti-inflammatory and antioxidant effects, and a decrease in estrogen levels exacerbates oxidative stress and systemic inflammation (46). Estrogen may enhance the protective effect of a low NLR in ALI, leading to a more significant association with prognosis in female patients.

Our research has a number of advantages. First of all, it is the first study to show that ALI has predictive significance for MAFLD prognosis. Second, the large, nationally representative cohort strengthens the generalizability of our findings. Third, our results’ robustness is reinforced by thorough comprehensive covariate adjustment and sensitivity analyses. However, the study has certain drawbacks. Because this was an observational study, a causal relationship between ALI and mortality cannot be established. Additionally, residual confounding factors cannot be entirely excluded. Thirdly, although sensitivity analyses expanding the study population through another.

MAFLD diagnostic criteria were performed, the high exclusion rate due to missing data may raise concerns about possible selection bias. Large-scale interventional research is required in the future to confirm these results and elucidate the causative mechanisms.

## 5 Conclusion

Our study provides an effective approach for predicting and managing patients with MAFLD. Low ALI levels were associated with poor prognosis, highlighting the utility of ALI in identifying populations at high risk of MAFLD and facilitating timely interventions. The observed L-shaped non-linear relationship shows that: when the indicators are below specific thresholds (all-cause mortality 71.48, cardiovascular mortality 68.54), a higher ALI was significantly associated with reduced mortality risks in MAFLD patients; however, once the thresholds are exceeded, the protective effect will stabilize. This underscores the importance of maintaining an optimal ALI level to improve survival outcomes in patients with MAFLD. Future cohort studies of ALI in MAFLD populations in other countries are needed. Explore intervention measures for ALI to promote more effective disease prevention and treatment.

## Data Availability

The datasets presented in this study can be found in online repositories. The names of the repository/repositories and accession number(s) can be found in the article/supplementary material.

## Glossary

ALI: Advanced lung cancer inflammation index
NAFLD: on-alcoholic fatty liver disease
MAFLD: Metabolic dysfunction-associated fatty liver disease
MASLD: metabolic dysfunction-associated steatotic liver disease
NHANES: National Health and Nutrition Examination Survey
T2DM: Type 2 diabetes mellitus
NDI: National Death Index
ICD-10: International Classification of Diseases, Tenth Revision
RCS: Restricted cubic spline
ROC: Receiver operating characteristic
AUC: Area under the curve
BMI: Body mass index
PIR: poverty-income ratio
HRs: hazard ratios
ALT: Alanine transaminase
AST: aspartate aminotransferase
HDL: high-density lipoprotein
TC: total cholesterol
TG: triglycerides
DM: diabetes mellitus
CVD: cardiovascular disease
HBP: high blood pressure
FLI: Fatty liver index
US-FLI: United States fatty liver index
PLR: platelet-to-lymphocyte ratio
SII: systemic immune-inflammation index
NLR: neutrophil-to-lymphocyte ratio
IL: interleukin
